# Metastasis of colorectal adenocarcinoma to the right ventricle in a young man: a case report

**DOI:** 10.11604/pamj.2023.44.32.37999

**Published:** 2023-01-17

**Authors:** Ahmed Badheeb, Faisal Ahmed, Yahya Alhosni, Mohamed Badheeb, Hamoud Obied, Islam Seada

**Affiliations:** 1 Department of Oncology, King Khalid Hospital, Najran, Saudi Arabia,; 2 Department of Medicine, Faculty of Medicine, Hadhramaut University, Hadhramout, Yemen,; 3 Urology Research Center, Al-Thora General Hospital, Department of Urology, School of Medicine, Ibb University of Medical Sciences, Ibb, Yemen,; 4 Department of Cardiology, King Khalid Hospital, Najran, Saudi Arabia,; 5 Department of General Medicine, King Khalid Hospital, Najran, Saudi Arabia,; 6 Department of Cardiac Surgery, King Khalid Hospital, Najran, Saudi Arabia,; 7 Department of Thoracic Surgery, King Khalid Hospital, Najran, Saudi Arabia

**Keywords:** Cardiac metastasis, colorectal cancer, right ventricle, case report

## Abstract

Cardiac cancers are exceedingly rare, mainly appearing as secondary cancers in patients with numerous systemic metastases, and are often detected by autopsies. Right ventricle (RV) metastasis from metastatic colorectal cancer (mCRC) is rarely reported in the literature. We report a 24-year-old man's case of mCRC, who developed positional variation in pulse rate while receiving the second cycle of chemotherapy. The echocardiography and chest computed tomography (CT) scan showed RV mass. The tumor was right-sided, and Kirsten rat sarcoma (KRAS) mutated based on that. He was treated with FOLFOX chemotherapy protocol plus bevacizumab and enoxaparin with an initial reduction in mass size. However, follow-up CT 9 months later demonstrated disease progression. For that, the chemotherapy was stopped, and the patient received palliative care. In conclusion, this case provides evidence that mCRC and RV location of metastasis can and do occur. In such a case, therapeutic intervention should be determined by weighing the benefits and risks.

## Introduction

Cancers involving the heart are more commonly metastatic than primary, with a very poor prognosis [1]. Although clinical recognition of cardiac metastasis from metastatic colorectal cancer (mCRC) is rare and continues to present a diagnostic and therapeutic challenge, this neoplasm should be considered among the broad differential of cardiac intracavitary masses [2,3].

Right ventricle (RV) metastasis from metastatic colorectal cancer (mCRC) is rarely reported in the literature [4,5]. Herein, we report a 24-year-old male with a case of mCRC who presented with positional variation in pulse rate and was diagnosed with an RV mass. In addition, we conducted a literature review on the clinical assessment and treatment of relevant cases.

## Patient and observation

**Patient information:** a 24-year-old single man, smoker, khat (Catha edulis) chewer, and soldier presented with abdominal pain, weight loss, and rectal bleeding in the last two months. The pain was generalized, severe and did not subside with medical treatment. He also mentioned a history of constipation and a change in bowel habits. The patient had no chronic medical conditions or family history of malignancy.

**Clinical findings:** the patient's vital signs were as follows (blood pressure: 110/70mmHg, respiratory rate: 14 respirations per minute, pulse rate: 91 (in supine position), 121 (in sitting) beats per minute). The chest and abdominal examinations were normal.

**Timeline:** the timeline of all events is mentioned in Table 1.

**Table 1:** events and progress of the disease

Events	Date
Initial symptoms	23/1/2022
Adenocarcinoma of rectosigmoid with liver metastasis	8/2/2022
Start chemotherapy	28/2/2022
Right ventricle metastasis	16/3/2022
Bowel obstruction and colostomy procedure (after cycle 3)	12/4/2022
KRAS mutation result and adding bevacizumab	05/5/2022
Follow-up CT scan	26/5/2022
Obstructive jaundice and first ERCP procedure	07/7/2022
Second ERCP procedure	17/7/2022
Final CT scan and end of the chemotherapy	13/7/2022
Death	13/9/2022

KRAS: Kirsten rat sarcoma; CT: computed tomography; ERCP: endoscopic retrograde cholangiopancreatography

**Figure 1:**
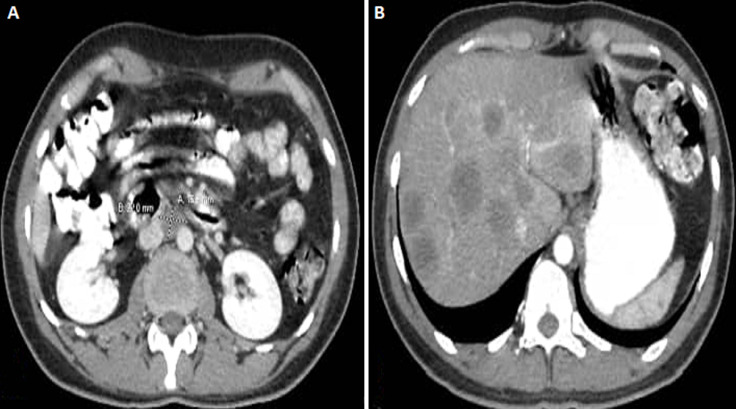
abdominal pelvic computed tomography scan showing: A) colonic mass; B) the liver metastasis

**Diagnostic assessment:** laboratory tests were significant for Carcinoembryonic Antigen (CEA) of 17 ng/mL (normal 0 to 2.5 ng/mL) with normal CA 19.9 of 7 ng/mL. Other laboratory tests were normal, including basic metabolic panel and renal and liver functions. The initial chest X-ray was unremarkable. Computed tomography (CT) scan of the abdomen and pelvis showed segmental significant circumferential mural thickening involving the sigmoid colon (about 6 x 1.4cm) and significant fat stranding. Another similar mass involves the distal rectum down to the anal verge (about 9 x 1.1 cm). A few prominent lymph nodes were seen within the mesorectum, para-aortic, and porta-hepatis, with the largest of the para-aortic group measuring approximately 17x22 mm (Figure 1A). Multiple hypodense lesions in the liver ranged from 5 mm to 37 mm (Figure 1B). The chest CT scan was normal. A colonoscopy revealed a mass located 15-17cm from the anal verge; the biopsy was taken and sent for histopathology result showed moderately differentiated adenocarcinoma of rectosigmoid (pT4N2M1) on February 2022.

**Figure 2:**
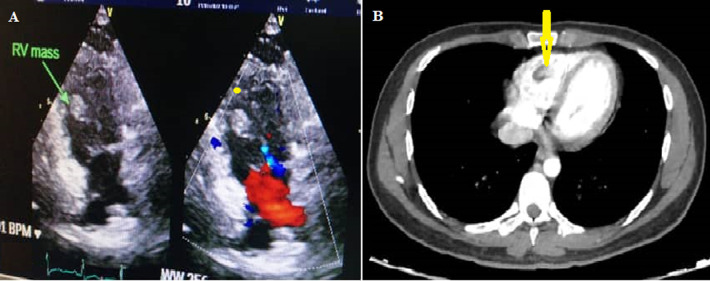
A) echocardiography showing the right ventricle mass (arrow); B) chest computed tomography scan showing the right ventricle mass (1.9 x 1 x 0.5 cm) (arrow)

**Therapeutic interventions:** the patient was diagnosed with mCRC (pT4N2M1) and treated with combination chemotherapy of 5-fluorouracil 400 mg/m^2^ bolus followed by 2400 mg/m^2^ as 46 hrs. infusion, leucovorin 20 mg/m^2^, oxaliplatin 85 mg/m^2^, every three weeks (FOLFOX regimen). Two weeks later, while receiving the second cycle of chemotherapy, we noticed positional variation in pulse rate (91 beats per minute in the supine position and 121 beats per minute in sitting). The electrocardiogram showed sinus rhythm. Echocardiography showed a filling defect within the RV measured about 19 x 10.5 mm, without pleural effusion or pneumothorax (Figure 2A). Cardiac magnetic resonance imaging (MRI) was impossible as the patient´s body was full of shrapnel due to previous exposure to a mortar blast. Chest CT scan showed a filling defect within the RV measuring about 19 x 10.5 mm, without lung mass, pleural effusion, or pneumothorax (Figure 2B). When the diagnosis of RV mass was made, a therapeutic dose of enoxaparin 1mg/kg subcutaneously every 12 hours was introduced.

**Figure 3:**
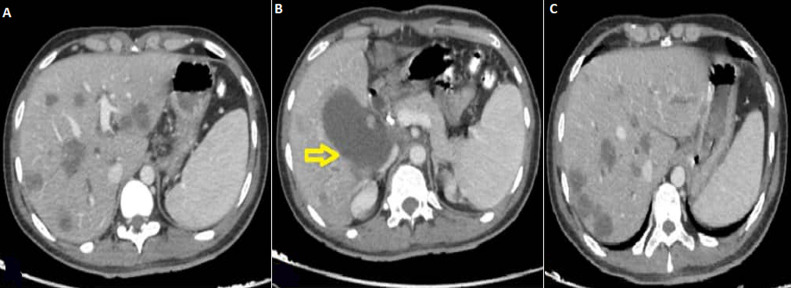
A) changes in metastatic liver lesions evaluated by computed tomography during chemotherapy at five months; B) abdominal computed tomography scan showing common bile duct dilatation (arrow); C) follow-up abdominal computed tomography at nine months showing no regression in mass size

**Follow-up and outcome:** the patient completed three cycles of FOLFOX chemotherapy with mixed responses, a drop of CEA from 17 ng/mL to 11 ng/mL, an almost normal level, and some radiological regression of liver lesions (Figure 3A). However, he developed bowel obstruction three days later. For that, a colostomy procedure was performed. When the result of the KRAS mutation was released as KRAS mutated, bevacizumab 5 mg/kg was added to chemotherapy from cycle 7. At this time, the patient developed jaundice. Abdominal CT scan showed a distended gallbladder measuring about 12 cm in long axis with no obvious wall thickening or adjacent fat stranding or free fluid (Figure 3B). The patient underwent endoscopic retrograde cholangiopancreatography (ERCP), which showed a proximal common bile duct (CBD) stricture of 2 cm. The patient underwent endoscopic sphincterotomy, and two plastic CBD stents were inserted with good bile drainage without complications. However, the patient developed jaundice again ten days later due to the down migration of stents. For that, he underwent a second ERCP with the change of CBD stents.

A nine-month follow-up CT scan showed disease progression (Figure 3C), with a rise of CEA from 11 ng/mL to 41 and CA 19.9 from 14 ng/mL to 19 ng/mL. Additionally, the RV mass increased in size within six months of anticoagulant therapy. For that, the chemotherapy was stopped, and the patient received palliative care. The patient ultimately died due to heart failure 2 months later.

**Patient perspective:** the patient was delighted with the level of care he received throughout therapy. Early in his suppurative treatment, he maintained social and functional engagement. The patient comprehended the final phase of his condition.

**Informed consent:** written informed consent was obtained from the patient for participation in our study.

## Discussion

We introduce a rare case of mCRC with liver and cardiac RV metastasis in young men treated with chemotherapy FOLFOX-bevacizumab regimen, plus enoxaparin, after confirmation of KRAS mutation. The patient had several events during chemotherapy, such as bowel obstruction and obstructive jaundice, that were successfully managed. The patient first showed a mixed response after five cycles of chemotherapy. However, he experienced an increase in tumor size (chemotherapy failure at seven cycles) and died due to heart failure two months later. Regardless, such cases remain extremely rare. Globally, colorectal cancer (CRC) represents the third most prevalent malignancy. Distant metastasis presents in almost 20% and 30% of cases at the time of diagnosis or during illness, respectively [4]. CRC metastasizes hematogenous, via lymphatics, or intracavitary spread, with the liver, lungs, and regional lymph nodes being the most involved structures [1].

More than 75% of primary cardiac tumors are benign, of which myxomas represent 50%, while sarcoma accounts for the remaining malignant cases. These tumors, however, are sporadic and diagnosed in only 0.001 - 0.28% of autopsy cases [6]. On the other hand, metastasis to the heart represents a unique metastatic pattern found in 2.3 - 18.3% of autopsy cases [6]. mCRC to the heart incidence has been reported variably in the literature. For instance, Bussani *et al*. reported cardiac metastasis in 9.1% of all malignant tumors [6]. Other studies reported an incidence of 1.4% to 7.2% [7]. Nevertheless, the actual incidence rate of cardiac metastasis may be underestimated, given the silent nature of such lesions [8].

Metastatic colorectal cancer (mCRC) has a median age of 70 years, ranging from 41 to 81 years, and predominantly affects males [2]. Our case was male and younger than the reported age period. Early-onset CRC generally has more aggressive characteristics with lymphovascular invasion, T3/T4 tumors, poor cell differentiation, and metastatic disease. In addition, young adults with CRC are often asymptomatic, and therefore diagnosis is often delayed. This delay in diagnosis lends itself to an advanced stage of disease at the time of diagnosis and, therefore, a worse prognosis [8].

The interval between the initial diagnosis of primary malignancy and the discovery of cardiac metastasis appears to be very wide, with controversy in the literature. While Sarfraz *et al*. reported that the interval appears to extend to 17 years after the initial diagnosis of CRC [2]. Others discovered this tumor within a short period or at the time of diagnosis, such as Mikami *et al*. (4 months), Choi *et al*. (at the time), and our case (3 months) [8,9]. Characteristics of reported mCRC patients with cardiac RV metastasis are summarized in Table 2 [1,3,4,9-18]. Radiologic investigations such as MRI, CT scan, and echocardiography might be helpful in the differential diagnosis of cardiac mass [8]. In our case, cardiac MRI was impossible as the patient´s body was full of shrapnel due to previous exposure to a mortar blast. Moreover, the CT scan and echocardiography were enough to reach the diagnosis.

**Table 2:** characteristics of colon cancers with cardiac ventricular metastasis

Case number	Year	Age*/ gender	Primary site of tumor	Time to cardiac metastasis	Tumor size (cm)	Treatment	Outcome
Woodayagiri *et al*.	2022	62/F	Descending colon	N/A	1.5 x 2.5	No treatment	Died
Butler *et al*.	2012	60/F	Rectosigmoid	19 years	Multiple masses	Surgery + chemotherapy	Still alive to 2011
Karabag *et al*.	2018	67/M	N/A	At the time	6 x 5	Surgery	Died after surgery
Elbatarny *et al*.	2019	59/M	Colon	17 years	5.2 x 3.9 x 2.9	Surgery	Still alive
Norell *et al*.	1984	60/F	Sigmoid	Three years	N/A	No treatment	Died
Mikami *et al*.	2015	76/F	Ascending colon	4 months	7 x 5	No treatment	Died after 7 months
Parravicini *et al*.	1993	45/M	Rectum	2 years	10.5 x 4 x 3.5	Surgery + chemotherapy	Died after 7 months
Lord *et al*.	1999	71/M	Rectum	3 years	N/A	No treatment	Died after 3 weeks
Pizzicannella *et al*.	2012	35/M	Colon	1 year	6.4 x 5 x 2.6	Chemotherapy	Died after a few months
Tsujii *et al*.	2017	76/F	Transverse colon	At the time	5.4 × 3.2 × 3.1	Chemotherapy	Still alive
Hiroi *et al*.	2020	71/M	Sigmoid	3 years	3	Chemotherapy	Still alive t0 2021
Tomiyama *et al*.	2021	71/M	Ascending colon	At the time	10 x 7	No treatment	Died after 3 months
Our case	2022	24/M	Rectosigmoid	4 months	1.9 x 1 x 0.5	Chemotherapy	Died after 11 months

* Age at primary diagnosis; N/A: not mentioned; F: female; M: male

Due to the rarity of CRC with cardiac metastases, surgery has not been studied extensively as a treatment approach. Although not usually advised for treating metastatic cardiac tumors, Koizumi *et al*. observed that obstructive and isolated lesions are particularly amenable to surgical therapy for symptom alleviation and life extension [5]. The incidence of cardiac involvement from mCRC is predicted to rise due to improved diagnostic techniques and a longer life expectancy. Further research is required to define the significance of surgical therapy in CRC cardiac metastasis. Our patient did not exhibit any cardiac symptoms, and anti-coagulants were initiated for his condition.

The treatment of mCRC with chemotherapy multiple therapy results in varying therapeutic responses; however, patients inevitably experience disease progression or recurrence. Choufani *et al*. described a patient with mCRC to the liver 16 months after treatment, who presented with abdominal distension and exertional dyspnea coupled with a right atrial mass, advancement of liver metastases, new signs of ascites, and pleural effusions. His right atrial mass and ascites disappeared entirely after he had four monthly doses of irinotecan. Nonetheless, successive CT scans revealed a partial return of the right atrial tumor and increased CEA levels. Ten months following the resumption of irinotecan, the patient's symptoms were well-controlled [19].

In comparison, a case described by Tsuji *et al*. featured an incidentally detected RV tumor with histopathology confirming the presence of wild-type KRAS. Surgeons considered the tumor unresectable. Following ten rounds of 5-fluorouracil, oxaliplatin, and panitumumab were administered to the patient, oxaliplatin was withheld due to neuropathy, and the patient was found to be in partial response after 12 courses (10 months after initial treatment). However, a follow-up CT after 15 cycles revealed increasing RV mass, and the patient chose palliative treatment but was still alive two years after the diagnosis [1]. In our case, while the initial CT scan mentioned a notable reduction in liver metastatic lesions size, the 9-month CT scan showed progressive disease, and the patient elected for palliative care; one of the possible explanations here is the KRAS mutation in our patient.

De la Fouchardière *et al*. described a patient with rectal carcinoma, persistently elevated CEA levels, and subsequent positron emission tomography (PET)/CT scan revealed a 6 x 3 cm tumor in the RV. With a palliative intent, the RV mass was resected and deemed histopathologically of rectal origin. A postoperative CT scan revealed the presence of a persistent intracardiac mass and pericardial effusion. After six cycles of FOLFIRINOX, the cardiac mass was found stable, and the pericardial effusion was reduced. The patient completed eleven FOLFOX cycles with no reported symptoms [20]. A similar case was reported by Sarfraz *et al*. who received a FOLFIRINOX/ bevacizumab for mCRC to the right ventricle, and interestingly, the mass showed reduction in size following chemotherapy [2].

## Conclusion

Cardiac metastasis of colon cancer is a rare entity. Patients with CRC should be evaluated for possible cardiac metastasis upon developing new cardiac-related symptoms or identifying new cardiac lesions in imaging. Currently, there are no standardized approaches to treatment in patients with cardiac metastases from CRCs. More research is needed to determine the best treatment strategy for this group of patients.
